# Towards Additive Manufacture of Functional, Spline-Based Morphometric Models of Healthy and Diseased Coronary Arteries: In Vitro Proof-of-Concept Using a Porcine Template

**DOI:** 10.3390/jfb9010015

**Published:** 2018-02-02

**Authors:** Rachel Jewkes, Hanna E. Burton, Daniel M. Espino

**Affiliations:** Department of Mechanical Engineering, University of Birmingham, Birmingham B15 2TT, UK; Rachel.jewkes@hotmail.co.uk (R.J.); HEM344@student.bham.ac.uk (H.E.B.)

**Keywords:** additive manufacture, CAD, coronary arteries, hemodynamics, models, morphometric, PolyDiMethylSiloxan (PDMS), rapid prototyping

## Abstract

The aim of this study is to assess the additive manufacture of morphometric models of healthy and diseased coronary arteries. Using a dissected porcine coronary artery, a model was developed with the use of computer aided engineering, with splines used to design arteries in health and disease. The model was altered to demonstrate four cases of stenosis displaying varying severity, based on published morphometric data available. Both an Objet Eden 250 printer and a Solidscape 3Z Pro printer were used in this analysis. A wax printed model was set into a flexible thermoplastic and was valuable for experimental testing with helical flow patterns observed in healthy models, dominating the distal LAD (left anterior descending) and left circumflex arteries. Recirculation zones were detected in all models, but were visibly larger in the stenosed cases. Resin models provide useful analytical tools for understanding the spatial relationships of blood vessels, and could be applied to preoperative planning techniques, but were not suitable for physical testing. In conclusion, it is feasible to develop blood vessel models enabling experimental work; further, through additive manufacture of bio-compatible materials, there is the possibility of manufacturing customized replacement arteries.

## 1. Introduction

Cardiovascular disease is the leading global cause of mortality, of which coronary artery disease has the highest mortality rate [1]. Coronary artery disease is an accumulation of lipid based plaques on the inner arterial walls resulting in occlusion [2,3]. This vascular occlusion can lead to hypoxia, impaired ventricular function, and acute myocardial infarction (i.e., a heart attack) [4]. The coronary arteries stem from the aorta, with the primary function of distributing oxygenated blood to the myocardium [5]. The left main coronary artery bifurcates into the left anterior descending (LAD) and left circumflex (LCX) arteries, supplying blood to the left ventricle, septum, and left atrium [6,7,8]. 

The bifurcation site of an artery, where one artery divides into two smaller arteries, is the most common location for plaque build-up and rupture [9,10]. Understanding the hemodynamic effects of stenosis is clinically important to aid rapid categorization of stenosis severity and diagnose atherosclerosis. The analysis of hemodynamics in the left coronary artery is a good example of blood flow, as the flow characteristics are commonplace in the rest of the arterial tree [11]. The left coronary artery also sees a high frequency of disease [2]. Pulsatile flow patterns and pressure gradients across bifurcations are useful research tools for designing vascular implants for coronary artery disease treatment [8,12,13] and to advance medical techniques for combatting atherosclerosis [8,14,15]. 

Additive manufacturing techniques can employ the use of three-dimensional (3D) printers, which could be employed to predict flow profiles within coronary arteries. It is equally as important to investigate healthy and diseased cases, and to evaluate the differences in flow patterns and hemodynamic parameters. Additive manufacture is particularly applicable to modelling blood vessels as complex geometries with diverse material properties which can be quickly and accurately printed to facilitate testing [16,17]. Utilizing 3D printing to build structures layer-by-layer from computer aided engineering files was initially developed with the intention of manufacturing parts or models for industries including automotive and aerospace engineering. The medical industry first used rapid prototyping techniques in 1990 to better understand spatial relationships in the human skull [14]. Additive manufacture techniques have significantly advanced; medical health professionals can combine data from computed tomography (CT) and magnetic resonance imaging (MRI) to enable patient specific additive manufacture in the assistance of diagnosis, surgical intervention, implants, and medical device developments [18].

Computationally and experimentally hemodynamic studies have the potential to inform clinical practice and disease. Comparative analyses of healthy and stenosed arteries has previously been carried out on human, porcine, and canine arteries, with the majority of these studies carried out on computationally reconstructed models [10,13,19,20,21,22,23] or in vivo [4,24,25,26,27,28]. Of the ex vivo experiments in the literature, most analyze flow characteristics in a nominally restricted tube due to the experimental difficulties in generating the complicated anatomy of blood vessels, which can limit conclusions [29,30,31,32,33,34,35]. In a study by Xu et al. investigating the accuracy of additive manufacture intracranial arteries from magnetic resonance angiography images, 87.5% of clinicians deemed the models useful for preoperative planning [17]. Accurate 3D models of human hearts have been fabricated in the past using silicone or paraffin wax casts of specimens obtained from autopsy [14]. However, these are very limited in comparison to the possible prototypes which can be additively manufactured, particularly with the use of additive manufacture, and inherently useful for clinical assessment and surgical planning. There are limited studies to date into the implications of different additive manufacture methods on pre-clinical testing of coronary arteries; specifically, the difference between rigid and pliable models of blood vessels. There are inherent benefits in considering deformable polymers such as PolyDiMethylSiloxan (PDMS) which include biocompatible grades for this potentially functional biomaterial. It is commonly used for the manufacture of biomedical devices both at the macro- and micro-scale [36,37,38].

The aim of this study is to assess the additive manufacture of morphometric models of healthy and diseased coronary arteries. Briefly, a Computer Aided Design (CAD) model for the primary bifurcation of the left main coronary artery has been generated based on a porcine heart and defined based on morphometric measurements in the literature through the use of mathematically defined splines. This process has been used to generate clinically relevant models which reflect four common cases of stenosis seen in human arteries, in addition to a healthy model. Prototypes manufactured using hard and soft (PDMS) printing materials have been compared. These prototypes have subsequently been assessed to establish the feasibility of experimental testing on both a hydrostatic and dynamic system.

## 2. Results

### 2.1. Artery Models

**Figure 1:**
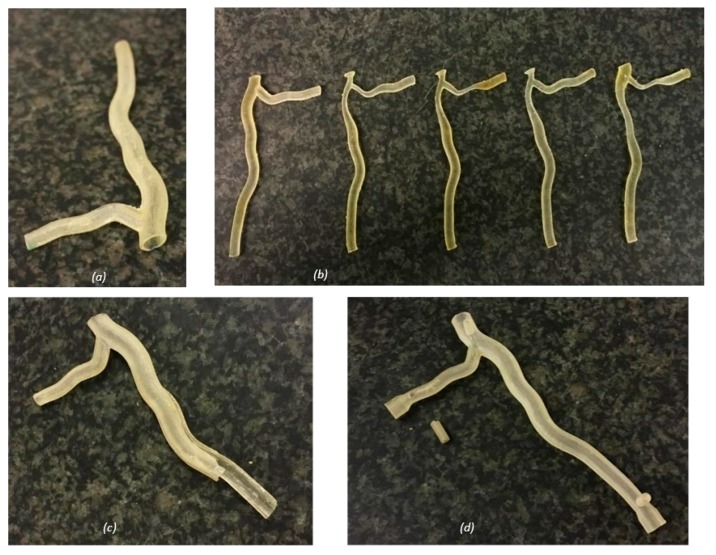
Printed resin models: (**a**) hollow healthy bifurcation; (**b**) anatomical solid models of the healthy and stenosed cases; (**c**) hollow artery model damage, with cracking along the length of the artery; and (**d**) hollow artery model with a connection via a small port (damaged during testing). Full design rationale and dimensions used to design the models are included in detail in Section 4.1.

Of the two printing methods used, the resin printer had a higher resolution, fabricating more anatomically accurate models (Figure 1). The run time of each printer was similar, within the region of three hours for each model. The material requirements for each resin model were small, and waste material was minimal. However, the printed resin models undertook damage during experimental testing, with cracks propagating along the length of the model (Figure 1c). Small connection ports were also broken off during testing, due to the brittle nature of the material (Figure 1d).

**Figure 2:**
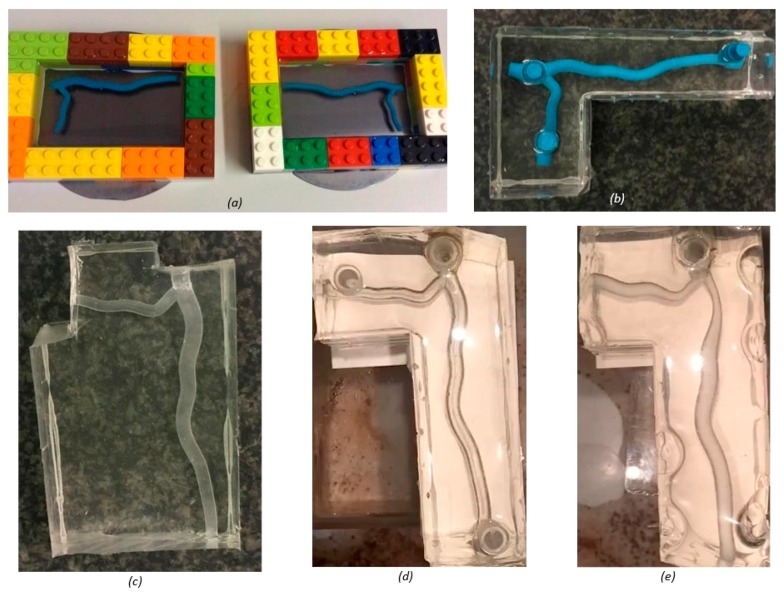
Wax printed PolyDiMethylSiloxan (PDMS) models: (**a**) half cross-section model set in PDMS using a Lego mold; (**b**) full cross-section model before wax was removed using acetone; (**c**) completed half cross-section model; (**d**) completed full cross-section model; and (**e**) defects seen in PDMS model of stenosis case one. Full design rationale and dimensions used to design the models are included in detail in Section 4.1.

To create the PDMS mold, an additional 24 h was required on top of printer run time to allow the thermoplastic to set; further time was needed to remove the wax using acetone (Figure 2). Of all print runs carried out, defects were only seen in the PDMS models, with large air bubbles inhibiting visibility (Figure 2e). Whilst less anatomically accurate than resin, the PDMS method proved superior for functional testing purposes. Table 1 summarizes the key differences in printing methods.

### 2.2. Flow Visualisation Under Hydrostatic Pressure

The cross-sectional model did offer optical benefits; flow characteristics in the middle of the artery could be seen, however, it prevented flow patterns from fully developing. Helical flow profiles were seen in the full cross-section model, whereas, flow appeared laminar in the half cross-section model.

**Figure 3:**
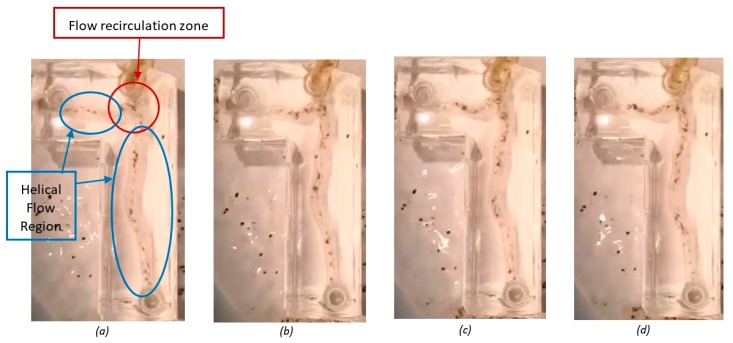
Sequential images taken from the flow visualization test of the healthy artery bifurcation taken at (**a**) 0.25 s; (**b**) 0.5 s; (**c**) 0.75 s; and (**d**) 1 s.

There was evidence of helical flow patterns as flow particles travelled down the length of the artery; vortices were present in the plane of symmetry. These vortices appeared clear and well defined, dominating the distal LAD and LCX artery segments. A section of flow recirculation was seen across the proximal left main coronary artery near the bifurcation and minimally into proximal the LAD and LCX arteries. The velocity of the particles reversed in this area. The division of flow was consistent across the bifurcation, as the particle density downstream of the bifurcation was predominately equal. Figure 3 shows four sequential images taken within a timeframe of 1 second. Qualitative observations during these experiments were consistent with recirculation of flow near sites of bifurcations. Thus, helical flow patterns might be expected; however, this would require further verification using quantitative flow visualization methods.

**Figure 4:**
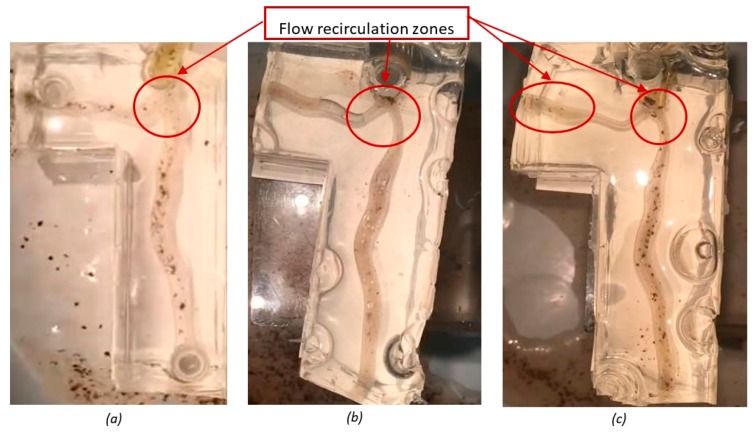
Static images taken from recordings of flow visualization tests: (**a**) healthy artery bifurcation (**b**) stenosis case one; and (**c**) stenosis case two.

Although little difference was seen between the flow patterns for stenosis cases one and two, there are comparable differences when considering the healthy bifurcation (Figure 4). Observations during testing of the particles within the fluid medium led to the qualitative inference that flow slowed across the stenotic regions, before increasing beyond the occlusion. A larger and more turbulent recirculation zone was seen upstream of the flow divider in both cases (Figure 4b,c), when compared to the healthy bifurcation.

One notable difference between stenosis the cases is the existence of a large area of back flow in the distal segment of the left circumflex in stenosis case two (Figure 4c), which is not as apparent in stenosis case one. A more defined helical flow pattern developing toward the distal end of the daughter arteries in the first stenosis case was seen in comparison to the second.

## 3. Discussion

### 3.1. Insights from Study

This study highlights the value of using additive manufacture to generate bio-inspired materials functionally structured to, for instance, enable pre-clinical assessment. A recent report by the Food and Drug Administration and the Medical Device Innovation Consortium highlights the need to better inform the development of medical devices through the increased use of computational modelling [39]. In this study, we have used spline-based Computer-Aided Design (CAD) to guide the design of diseased coronary arteries, subsequently 3D printed. The definition of parameters which describe anatomy is somewhat analogous to a recent morphometry based toolbox generated for the spine [40]; though, in our current study, disease has been defined too. The refinement of an accessible additively manufactured model can be applied to countless different blood vessels with complementarity to: development of stents; diagnosis of stenosis severity and surgical intervention requirements; patient specific blood flow modelling [41,42] or validation of computational models [43,44]; and improvement in the results of clinical treatment through detailed preoperative planning [45]. As there are biocompatible grades of PDMS [46], any 3D printed constructs hold potential for translation into clinical practice: patient specific implants [47], but for soft tissues.

The usefulness of the accurate resin anatomical models has been reflected in literature; commonly stereolithography is used in cranio-maxillofacial surgery to assist detailed pre-operative planning. The risk of complications is reduced where additive manufacture is used in medicine, due to the ‘touch to comprehend’ interactions surgeons gain [48]. Stereolithography produces transparent models, which in costly cases can be produced in multiple colors to identify different anatomical features [18]. Soft tissue models for cranio-maxillofacial surgery have also been made in the past using silicone [49], yet there is still scope for further development in the additive manufacture of synthetic arteries.

The mechanical properties of arteries have a significant effect on the flow parameters generated and, thus, should be considered when developing arterial models. An infinite pulse wave velocity is possible in a rigid model, but not in an elastic model [50]. The mean flow has also been shown to be up to 10% greater in elastic models than rigid models of the femoral artery [51]. In a comparative study, it is also seen that the size and velocity of recirculation areas are significantly increased in elastic models of a right carotid bifurcation [52]. Therefore, the elastic properties of the PDMS models developed provide potential for results superior to rigid constructs (e.g., [4,33,34]). Comparing the Young’s modulus, the accepted range for human arteries is between 0.3 and 5.5 MPa [53,54], A CES Edupack (Granta Design Ltd., Cambridge, UK) examination of PDMS material properties shows that its Young’s modulus lies within the range of human tissue at 0.36 and 0.87 MPa. Cardiovascular tissues function within a dynamic environment, and can be characterized according to dynamic viscoelasticity, i.e., storage and loss moduli [55,56,57]. Coronary arteries have a storage modulus in the range of 14–25 MPa storage modulus and around 2 MPa for the loss modulus [58]; currently, the authors are unaware of equivalent measurements for PDMS, but such data would be valuable for bio-mimicking constructs and/or replacement constructs.

In this study, a simple flow-visualization technique was used, as proof-of-concept. However, more advanced techniques could be used in conjunction with seeding particles. Although there are limitations with the technique presented in this study, a qualitative and inexpensive method was used to prove the printing method had compatibility with hemodynamic testing systems. Further, the flow patterns identified here match results from existing physical tests on coronary artery models [23,59,60,61,62]. In our study, the existence of helical flow was predicted in coronary arteries. However, a more extensive computational study of four realistic and eight simulated left coronary arteries focusing on the region chosen in this study has been analyzed by others. No helical flow patterns were reported as a result of flow pathline reconstruction, which is seen as a limitation of computational methods [63]. The area of flow separation seen in all artery models in the proximal left main coronary artery was expected, where flow patterns took the form of eddies and unstable vortices [33]. A recirculation zone close to the flow divider has been seen in comparative studies of coronary arteries, using both an idealized physical geometry [64] and a Fluid-Structure Interaction study from a CT scan [21].

Comparing the healthy and occluded cases, the larger recirculation zone seen downstream of the stenosis is typical. A computational fluid dynamics analysis of two-dimensional and 3D arteries conducted with plaque burdens of 30%, 60%, and 80% predicted an increase in recirculation zone as percent stenosis increases [65]. It was also noticed that the flow patterns appeared to be much simpler and easier to distinguish in the healthy cases than when considering disease. Physical testing of five arterial tree molds prepared post mortem revealed that bifurcation sites were the most common location for spiral flow patterns to be seen, and that flow patterns were far simpler to identify in healthy bifurcation as the larger variations in anatomical structure in diseased arteries caused a range of different effects [62].

### 3.2. Limitations and Future Horizons

The coronary arteries lie within the surface of the heart, undergoing large dynamic changes over the course of the cardiac cycle. Olson et al. concluded that curvature is the dominant governing effect influencing flow phenomena [66]. This study has aligned the artery models within a two-dimensional (2D) plane, ignoring the effects of curvature. However, coronary flow occurs mainly during diastole, where skew of the velocity profile due to heart curvature has least effect [64]. A clinician wanting to investigate a patient’s artery to aid diagnosis or preoperative planning would require specific geometry for assessment; thus, the splines assessed in this study would need to be combined with additional definitions for curvature. The process could, in addition, be fully automated, as demonstrated recently by Lavecchia et al. [40].

The flow visualization tests in this study have been carried out using a hydrostatic system, which is not representative of the pulsatile nature of cardiovascular flow. Normal waveforms for the arteries during diastole are determined to be of relatively constant forward velocity [67,68]; therefore, this format is representative for at least one half of the cardiac cycle. The models developed in this study for hydrostatic testing have also been successfully integrated into a pulsatile system, proving their potential for use in future research. The post-processing of data recorded could then provide graphical representation of the pressure gradient through the cardiac cycle, which could further be used in the validation of computational models, and to develop procedures to quickly and non-invasively diagnose coronary artery disease. Furthermore, computational models can in-build patient specific boundary conditions for modelling [69,70,71,72].

As explained above, a hydrostatic system was used for these initial experiments. This means that quantitative measurements of pressure and flow would be limited as regards direct comparison to a physiological artery. Instead, flow was assessed purely qualitatively and based on observations of particles flowing suspended within the fluid, during flow through the artery structure. Given the proof-of-concept demonstrated in this study, future quantitative studies would now be warranted which measure flow velocity and orientation (e.g., using particle image velocimetry or laser doppler velocimetry). Further, the fluid used in this study was water; given that hydrostatic pressure is independent of the fluid viscosity, it has not altered the results obtained for the pressures used during experimentation. However, hydrodynamic experimentation will require the use of a fluid with viscosity which mimics that of blood.

**Figure 5:**
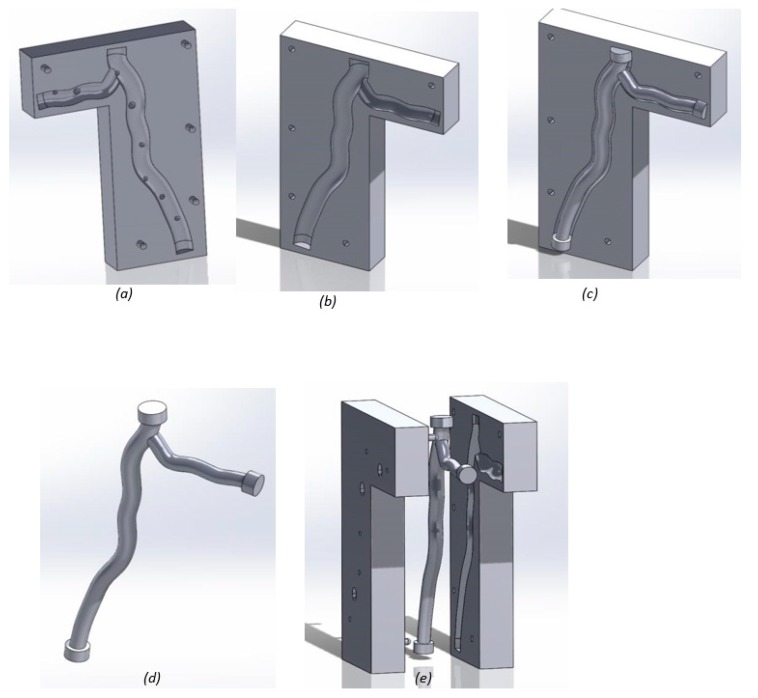
Mold design for future research:(**a**) mold top with pouring holes and air outlets printed using the resin printer; (**b**) mold base printed using the resin printer (**c**) inside mold inserted into the cavity in the mold base; (**d**) inside mold printed using the wax printer; and (**e**) three mold components demonstrating a potential fit.

The authors believe that it is feasible to combine an automated spline and curvature model with an automated process for model development based on a few input parameters. This presents the possibility of not only generating in vitro analogues, but with a biocompatible grade of PDMS, it also opens up the potential for replacement of natural soft connective tissues. In the case of coronary arteries, this may also require additional parameters such as a bio-inspired surface roughness [58,73]. Importantly, it is feasible to manufacture the surface roughness of PDMS constructs in the laboratory [74]. The question then remains whether the full process could be upscaled, or whether there are additional barriers to an industrial uptake [75] and clinical implementation. The first step in this process is producing a pliable construct with a thickness comparable to a real artery; the two investigated printing methods could be combined to create a mold such that the PDMS prototype has the wall thickness of an artery (Figure 5). As the material properties of PDMS [76] appear to lie in a range comparable to that of coronary arteries [58], this provides much promise towards future replacement with a functional biomaterial. The generation of fully biomimetic additively manufactured constructs would of course require further development of further physico-chemical and biological characteristics; in the mean-time, it is important to aim to develop materials and constructs which may provide realistic alternative to current clinical practice (be it metal stents, or use of arteries which are not part of the coronary circulation). Any clinical implementation may also require the inclusion of the underlying heart itself, which is known to be a dominant factor affecting flow [77]. While this study has focused on the design a coronary artery segment, and the potential for designing disease (i.e., potential uses for experimental studies), incorporating curvature into geometric models as a controllable variable is feasible [40] and is not a barrier for additive manufacture.

## 4. Materials and Methods

### 4.1. Computer Aided Engineering Model

#### 4.1.1. Healthy Left Coronary Artery Bifurcation

**Figure 6:**
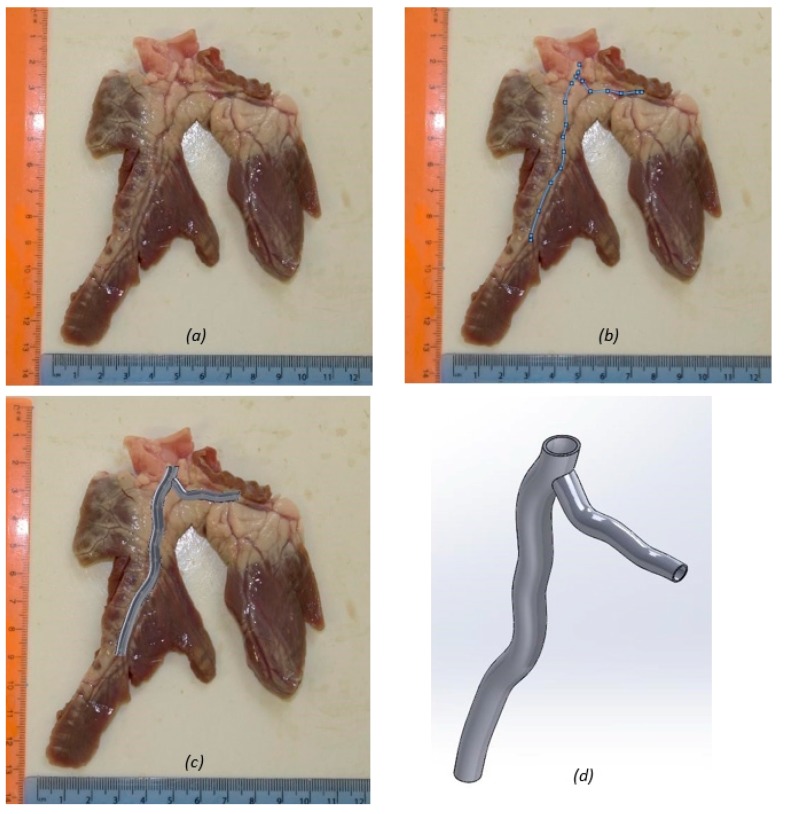
Image based modelling of the coronary artery: (**a**) dissected coronary artery with rulers used for scale; (**b**) spline governing the artery model; (**c**) healthy artery model superimposed on the porcine coronary artery specimen; and (**d**) healthy artery model.

The initial Computer Aided Engineering (CAE) Model was developed from the coronary artery of a healthy porcine heart, acquired from a supplier (Fresh Tissue Supplies, Horsham, UK). The coronary artery from the porcine heart was dissected and provided a scaled image of the first bifurcation (Figure 6). The image was then used to create a geometrically accurate spline shape from which to build the artery model (Figure 6). Whilst the model uses a subject specific shape, the lumen dimensions reflect an average human coronary artery bifurcation taken from clinical literature [8,78]. The CAD platform used was SolidWorks (Solidworks 64, Dassault Systémes, Vélizy-Villacoublay, France). The initial model was developed as a thin walled hollow tube (Figure 6), using the loft function to specify the inner lumen dimensions at points along the artery.

For a right dominant heart, the left main coronary artery measured 4.5 ± 0.5 mm in diameter with the proximal LAD measuring 3.7 ± 0.4 mm. This model (Figure 6d) ends at arterial segments C1 and L1, the proximal segments of the circumflex and anterior descending arteries, respectively, where diameters were 3.4 ± 0.5 mm and 3.6 ± 0.5 mm. These diameters were obtained from a study by Dodge et al. [78] detailing normal lumen diameters of a healthy human heart found from 83 clinical catheterization studies. Patients were chosen only where completely smooth lumen borders were detected and thus they were deemed free of atherosclerosis. The lumen diameters were normalized for gender, and for measurements taken at systole or diastole. This is important for the development of the physical model to ensure clinical relevance, despite being a static snapshot of the artery during its operation. The artery was initially built with a wall thickness of 0.68 mm. This dimension was calculated by adding the normal average of the intima, media and adventitia thicknesses; 80 μm, 200 μm, and 400 μm, respectively [8].

#### 4.1.2. Designing Disease

**Figure 7:**
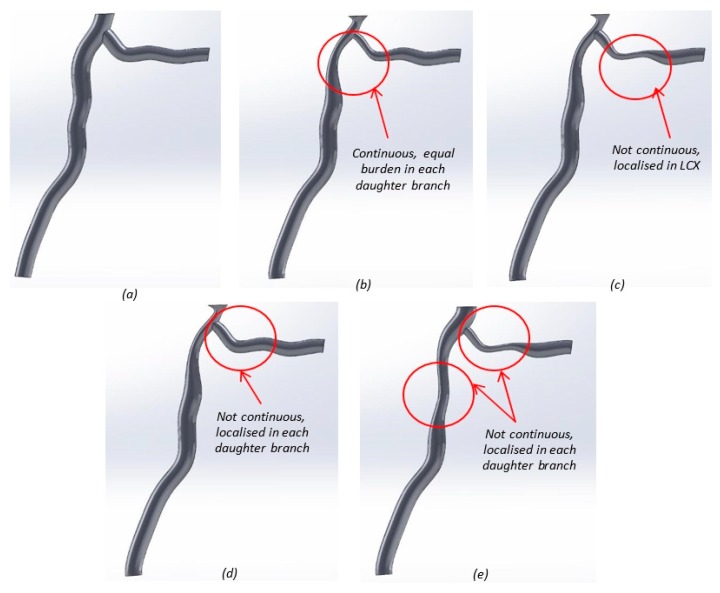
Solidworks models for the healthy and stenosed arteries: (**a**) healthy; (**b**) stenosis case 1; (**c**) stenosis case 2; (**d**) stenosis case 3; and (**e**) stenosis case 4.

Four general cases of stenosis were composed (Figure 7) to examine the hemodynamic effects of atherosclerosis. Although there are limitations due to the small size and changing nature of coronary plaques [79], plaque location and percent stenosis were chosen in each case for how often they were seen in a study of 140 human coronary artery angiograms, conducted by Oviedo et al. (Table 2) [80]. Plaque locations were categorized using intravascular ultrasound data and an edge detecting algorithm. Percent stenosis is a method used for clinically diagnosing the severity of coronary artery disease [11]. Stenosis is referred to as percent occlusion either by diameter or area. Here, diameter was used, governed by Equation (1), where *D*_1_ and *D*_2_ are the healthy and occluded diameters respectively.

(1) $$Stenosis\;\left(\%\right)=\left(\frac{D_{1}-D_{2}}{D_{1}}\right)\times 100$$

**Figure 8:**
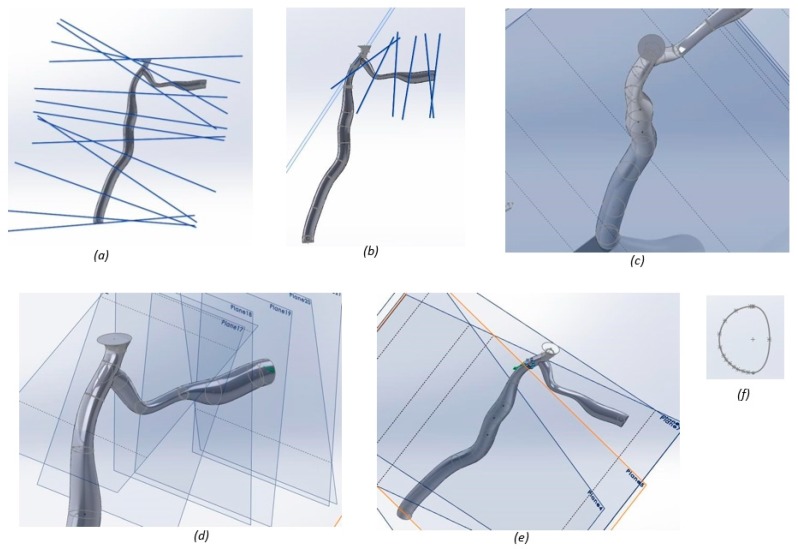
Model set-up for stenosis case 2: (**a**) planes used to govern the geometry for the left anterior descending (LAD); (**b**) planes used to govern the geometry for the left circumflex (LCX) artery; (**c**) geometric constraints for the LAD artery; (**d**) geometric constraints for the LCX artery; and (**e**) constraint to the lateral site plaque; (**f**) cross section of the artery.

To model disease cases, each stenosed artery was divided into planes located at the bending point of the spline (Figure 8). On each of these planes, the diameter of the artery was input, enabling the length of the stenosed section to be controlled for each case (Figure 8). Stenosis is not always evenly displaced around the inner lumen wall of the artery, and can be categorized as a lateral, myocardium, or pericardium site plaque burden [80]. Where plaque is not diffuse (i.e., circumferentially located), as in cases two and four, lateral site burden was chosen, as this occurs most frequently [80]. Lateral site burden was governed using blocks in SolidWorks, which were scaled in each instance (Figure 8). Table 2 shows the differences in plaque location for each stenosis case.

### 4.2. Additive Manufacture

#### 4.2.1. Printing Preparation

Two binder jetting printing methods were used in this project, and subsequently evaluated. Both hard resin and soft models were fabricated, detailed in Section 4.2.2 and Section 4.2.3, respectively. For both printing methods, a final SolidWorks part file was created from the associated assemblies. The part file was then converted into tetrahedral elements and saved as a STereo-Lithography (STL) file format, compatible with both printers.

#### 4.2.2. Resin Printer

The first printing method used an Objet Eden 250 printer (Stratrasys Ltd., Eden Prairie, MN, USA), printing in FullCure^®^ 720 photopolymer resin, with FullCure^®^ 705 photopolymer gel-like support material (both from Stratrasys Ltd., Eden Prairie, MN, USA) [81]. The resin printer produced a positive model, originally printed with no infrastructure for attachment to the proposed flow system, but with a hollow structure of wall thickness 0.68 mm. A second iteration was printed with added wall thickness, wall pressure taps, and porting compatible with flexible tubing to facilitate attachment to the flow system. Solid prototypes of each of the healthy and stenosed cases were printed as demonstrative physical models.

#### 4.2.3. Wax Printer

The second printer used was a Solidscape 3Z Pro 3D Printer (Solidscape Inc., Merrimack, NH, USA) printing in 3Z^®^ Model, an organic wax-like compound, and 3Z^®^ Support wax (both from Solidscape Inc., Merrimack, NH, USA) [82]. Once printed, the wax model was set into a flexible thermoplastic, PDMS. The 3Z^®^ Model material was then melted out using a water bath and acetone. The PDMS method created a negative mold where the cavity matched the artery dimensions. A ratio of 10:1 was used for the pre-polymer to catalyzer preparation. Both a half and a full cross-sectional model were printed to assess the superior method for particle visualization. The thermoplastic was chosen for its fully transparent nature, and its ability to easily set. It was tested for shrinkage from acetone and superglue by measuring the dimensions of a small sample of PDMS using a digital Vernier caliper. The sample was measured, before being left exposed to each substance for a period of 24 h, after which it was measured again. Both substances had a negligible effect on the PDMS sample. A full artery model was printed with inbuilt ports for compatibility with a pressure transducer to measure pressure gradients, and for use in flow visualization testing. This printing method was repeated for the printing of stenosis cases 1 and 2, which were chosen as representative models of a 50% and 40% stenosed artery.

### 4.3. Hydrostatic System Setup

The 3D printed models were attached to a steady state fluid flow system using plastic tubing, simple two-way valves, and a pressure head to simulate arterial pressure. An average blood pressure of 100 mmHg (1300 Pa) was used, which equates to a pressure head of 1.36 m. The pressure head was calculated using the pressure, density of liquid, and acceleration due to gravity. Filling an open syringe to the required height, and using flexible plastic tubing cut to length achieved the required pressure head. The PDMS model was then attached to the static system. Flow visualization was carried out using the hydrostatic system to assess the feasibility of particle imaging through each model, establishing how easily particles and flow patterns could be identified. All tests were filmed using an iPhone 6S (Apple Inc., Cupertino, CA, USA), filming in 720 pixels (vertically) at 240 frames per second, and repeated on all PDMS models.

### 4.4. Pulsatile Displacement System Setup

**Figure 9:**
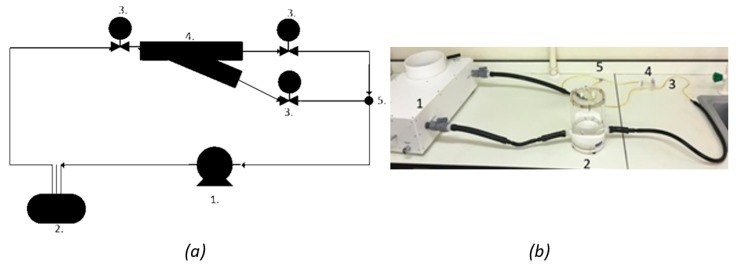
Suggested hydrodynamic flow system for future experimental work, including: (**a**) Pulsatile flow system schematic; and (**b**) pulsatile flow system laboratory. The proposed setup, as labelled in both (**a**,**b**) includes: **1**. a pulsatile displacement pump which provides a dynamic pressure wave to the PDMS artery model within set-up system; **2**. a compliance chamber to provide a back pressure to the artery, ensuring appropriate boundary conditions are applied; **3**. the use of two-way isolation valves which should be compatible with a transient pressure transducer; and **4**. PDMS model attached to the flow system via tubing; **5**. a junction to join the flow after the artery bifurcation.

The 3D printed models were set up on a pulsatile system to enable testing, whereby the system pressure matches the dynamic pressure of a human heartbeat. A system setup was used (Figure 9), similar to dynamic testing on physical coronary artery models in the literature [12,83,84]. The pump used was a PD-0750 pulsatile displacement pump (1) (BDC Laboratories, Wheat Ridge, CO, USA), with a pressure range of 80 to 120 mmHg, and an adjustable mean pressure of 100 mmHg. The fluid reservoir applied a backpressure to the system (2). Using this setup, pressure measurements could be taken by attaching a pressure transducer to the valves (3) either side of the bifurcation model (4). Preliminary experiments were performed to demonstrate compatibility, however, analysis of changes in flow were focused on the hydrostatic system described in Section 4.3.

## 5. Conclusions

The development of additive manufacturing processes, compatible with computational techniques, including those which can be automated for clinical efficiency, hold much promise for patient-specific medical treatment. In our study, we have successfully demonstrated a pre-clinical application of generating PDMS-based in vitro models for functional experimentation. The future horizons include the feasibility to print healthy and assess degenerate arteries through the control of geometric variables. Ultimately, proof of concept of the prototyping method has demonstrated the feasibility of such a process.

Ultimately, a proposed avenue for developing bio-inspired arteries is suggested, which utilizes additive manufacturing. The 3D printing technique employed in this study, to generate the PDMS models, first prints a wax model around (or within) which PDMS is set; the potential adaptation of this technique may well enable replication of the artery thickness during the manufacture procedure. Combined with a spline-based design process, this could result in the use of additive manufacture to enable the generation of subject-specific arteries using a biocompatible material.

## Figures and Tables

**Table 1:** Printer comparison.

	Objet Eden 250	Solidscape 3Z Pro
Model Material	Fullcure^®^ 720	3Z Model
Support Material	Fullcure^®^ 705	3Z Support
Printer Type	Polymer jetting technology	Drop-on-Demand Ink-Jetting technology
Layer Thickness (μm)	16	82
Time per Model (hours)	3	30
Defects in Printed Model	None	Air bubbles trapped in thermoplastic
Material Demand per Model	Minimal resin used for each model	Minimal wax used for each model High PDMS demand for each model
Compatibility with System	Breakages prevented testing	Fully compatible, enabling flexible tubing to be glued or inserted into infrastructure
Anatomical Accuracy	Very good	Good
Experimental Testing Value	Nil	Extremely useful

**Table 2:** Details of geometry for stenosis cases 1–4 [80].

	Case 1	Case 2	Case 3	Case 4
Percent Stenosis (%)	50	40	40	40
Plaque Location	Continuous from left main coronary artery into LAD and LCX	Continuous from left main coronary artery into LAD Localised in LCX	Continuous from left main coronary artery into LAD None in LCX	Not continuous from left main coronary artery Localised in LAD and LCX
Plaque Distribution	Diffuse	Diffuse Lateral	Diffuse	Lateral
Recurrence in Humans	62%	14%	14%	2%
